# Perspectives of Palestinian Healthcare Workers on Factors Affecting the Families’ Acceptance of News of Death: A Cross-Sectional Study

**DOI:** 10.7759/cureus.39001

**Published:** 2023-05-14

**Authors:** Oqab Jabali, Abdalhakim R. M. Shubietah, Mahfouz Ktaifan, Zaid Zakaria, Haytham Abumohsen

**Affiliations:** 1 Research, An-Najah National University, Language Center, Faculty of Humanities, Nablus, PSE; 2 Medicine and Surgery, Darwish Nazzal Government Hospital, Palestinian Ministry of Health, Qalqilya, PSE; 3 Research, An-Najah National University, College of Medicine and Health Siences, Department of Medicine, Nablus, PSE; 4 Medicine and Surgery, Palestinian Ministry of Health, Rafidia Government Surgical Hospital, Nablus, PSE; 5 Medicine and Surgery, Tubas Government Hospital,Palestinian Ministry of Health, Tubas, PSE

**Keywords:** healthcare providers, spikes, death reporting, breaking bad news, death acceptance

## Abstract

Introduction

In a healthcare setting, communication is essential for every aspect of care. The ability to break bad news to patients and families is one of the most crucial talents in a medical professional's communication toolkit. This study aims to investigate the factors affecting the family’s acceptance of death news in Palestinian medical facilities.

Methods

A survey was constructed and distributed to participants through Palestinian medical social media groups. Palestinian medical health professionals who had reported at least one death (N=136) were included. Associations and correlations were calculated. P-values of < 0.05 were considered significant.

Results

We found that death is more likely to be accepted by the family if it’s reported by an experienced staff member (p-value= 0.031) or a member who was involved in the cardiopulmonary resuscitation (CPR) of the deceased person (Adjusted odds ratio (AOR) = 19.335, p-value = 0.046). The medical ward staff is also more likely to achieve family acceptance (AOR = 6.857, p-value= 0.020). However, no evidence was found to support the claim that adhering to the SPIKES model increases the likelihood of family acceptance of death news (p-value= 0.102). Death of young people and unexpected death are less likely to be accepted (p-value < 0.05).

Conclusion

Families are less likely to accept unexpected death or the death of young members. Thus, reporting such deaths (mostly in the emergency department) should be done with greater care. We suggest letting experienced staff members or those who were involved in CPR report the death news in such situations.

## Introduction

Medical practitioners frequently have to converse with patients and their relatives in settings like hospital departments, accident venues, or people's houses. In these contexts, it is more common for rapport building to take place concurrently rather than successively [1]. In a healthcare setting, communication is essential for every aspect of care, including the content of information and communication style [2]. Regardless of the provider, patient-centered communication is important for providing high-quality medical care [3].

Delivering bad news causes physiological stress in patients, family members, and medical professionals [4]. One of the essential skills in a medical professional's communication repertoire is their ability to deliver bad news to patients and their families [5]. To effectively communicate bad news to patients and their families, physicians and other healthcare professionals must have a certain skill level [6]. In the past, little information was available on handling these extremely delicate situations [7]. Medical practitioners relied on their own experience instead of any training they may have received [8]. Recently, several protocols and techniques have evolved to improve the outcomes of breaking bad news; these include the SPIKES [9], BREAKS [10], and ABCDE [11] protocols. These protocols positively impacted both the patients and the care providers [9-11]. It was reported that medical practitioners who have received adequate training in this area would be better equipped to manage the difficult task of delivering bad news [12].

Communication issues have repeatedly been identified as a major obstacle in providing support to people during times of death and grief [13]. The acquisition of medical knowledge and practical skills is frequently prioritized over improving communication skills [14]. Medical professionals endure stress and discomfort as a result of their lack of experience and training in conveying bad news, particularly when it comes to sudden and unexpected death [15-17]. Medical education is woefully weak in this area [17]. Those in charge of communication must be aware that a sensitive attitude is crucial as it is capable of containing a person's reaction.

Based on the attachment theory, people are perceived as helpful and willing, lovable, and deserving of help [18]. The attachment system, which is automatically triggered in times of fear, anxiety, and exhaustion, is thought to act as a master regulator to lessen anxiety in interpersonal relationships (for example: between medical staff and patients and their relatives) by promoting connection to others [19]. As death is a topic that medical staff tend to avoid discussing openly [13], the current study relies on the attachment theory as it helps understand the perspectives of Palestinian healthcare professionals' on how medical professionals view families' acceptance of death news.

In Palestine, one of the public health concerns is the high incidence of workplace violence for healthcare providers (physical and emotional) [20-22]. Miscommunication was considered the most important factor provoking violent acts from patients and their relatives, especially in breaking bad news [20,21]. These studies supported evaluating and improving the communication skills of Palestinian healthcare professionals [20-22]. To our knowledge, this is the first study conducted in Palestine that describes the knowledge and use of breaking bad news protocols. In addition, the current study aims to investigate the factors (positively and negatively) impacting the family's acceptance of death news in the Palestinian medical facilities as well as the behaviors, strategies, and dynamics involved in breaking death news. The main areas of investigation are the experience of communicating death news and the level of acceptance by the decedent's family members. The study also seeks to determine whether there are any more suitable approaches or behaviors to take into account when it comes to breaking the news of a death and exploring the vulnerable groups based on gender, age, cause of death, the area in which death happened, and other factors. 

## Materials and methods

Study design, setting, and population

A cross-sectional survey was conducted to investigate and compare the medical staff's perception of how families accept the death news in Palestinian medical facilities, and to explore ways and approaches of reporting this news effectively. We selected participants by publishing/sending posts, emails, and direct messages through the common medical Palestinian social media platforms and groups, and then collected data using an online survey from 1st December 2022 to 28th February 2023. Only medical health professionals from Palestine who had at least reported death once (N=136) were included. In contrast, participants who turned out to be from outside Palestine (N=3) or who had not reported death at all (N =7) were excluded. As our survey was done via an online questionnaire, where you were required to answer all important questions, our results have no missing data. However, if a participant's response did not correspond to one of the choices, the "other" option was offered. As a result, we included all available data.

Operational Definitions

Sociodemographic data (age and gender) of the participant and deceased person, occupational data, and other questions about the death situation were collected through multiple-answer questions. Some questions allowed choosing multiple answers, while most were single-answer questions. If the participant's response was not of the options, it was recorded as "other". 

**Figure 1:**
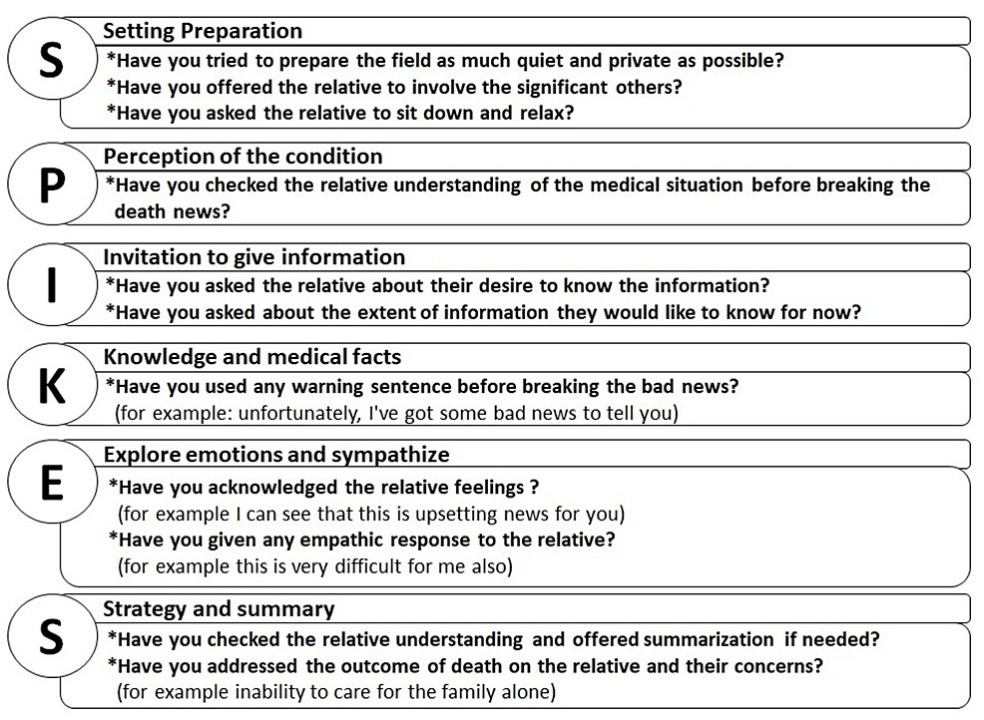
Components and questions used to assess the use of SPIKES protocol in the reported death The letters in the circles are the letters of the SPKIES protocol abbreviation. The corresponding upper box indicates the component which this letter represents, while the lower one indicates the questions used in our study to assess that component. Note: this figure is an original figure; the content of the table is obtained from the SPIKES protocol mentioned in the methodology section.

The participants were asked about the use of the SPIKES protocol components in the reported death using questions and expressions imported from the protocol itself [9] (Figure 1). A point was given for each component if the participant addressed any of the questions related to it, after which the total points were calculated. Participants who scored zero points (the minimum) didn't address any component of the SPIKES protocol, while those who scored six points (the maximum) addressed all the protocol components. 

The questionnaire was clearly explained in both languages (Arabic and English) and was checked by a medical doctor and an assistant professor in English. At the end of the survey, the participants were asked to double-check the answers, and if there was a mistake, they were allowed to correct it. After submission, the answers were exported to Excel.

Data analysis

The results were analyzed through Statistics Package for Social Sciences (SPSS. Version 22). We used descriptive statistics to characterize the sample. The Chi-square test was used to estimate the statistically significant differences between categorical variables, and the Pearson correlation coefficient was used to assess the difference between continuous variables. Binary logistic regression analysis models were conducted to determine the association between family acceptance of death and the other characteristics of the participants and the deceased. Adjusted odds ratios (AORs) and their 95% confidence intervals (CIs) were used as indicators of levels of association, and a p-value <0.05 was considered statistically significant. 

Ethical considerations

The Institutional Review Board (IRB) of the An-Najah National University (ANNU) issued approval (MED.DEC.2022.12) for this study. All personal and medical information was collected and analyzed confidentially.

## Results

Demographic characteristics and perception of how families accept the news of the death

The study participants' sociodemographic, occupational, and other characteristics (N=136) are presented below (Tables 1, 2). The mean age of participants was 30.51 ± 8.35, and the percentage of males (59.6%) was higher than females. Most participants were general practitioners (37.5%), and a large number worked in the emergency ward (29.4%). The participants were involved in cardiopulmonary resuscitation (CPR) in the majority of the death cases (52.2%) and expressed sadness when the patient passed away (66.9%). Only 21.3% of the participants had received training on the SPIKES protocol, and the majority of the participants (76.6%) had reported deaths before. In 66.9% of the situations, participants used a scenario to break the death news instead of directly reporting it, and the mean SPIKES score was 2.12 ± 1.63.

**Table 1:** Sociodemographic, occupational, and other characteristics of study participants Abbreviations: SD: standard deviation, CPR: cardiopulmonary resuscitation, Min: Minimum, Max: Maximum. * SPIKES, ABCDE, BREAKS are global protocol names for breaking bad news.

Variable	Mean (SD)	Min – Max
Participants age	30.51 (8.35)	20 - 78
Participant clinical experience (years)	5.932 (6.32)	0.3 - 25.0
Number of times participants reported death	22.19 (29.88)	1 – 150
Participant SPIKES score*	2.12 (1.63)	0 – 6

**Table 2:** Sociodemographic, occupational, and other characteristics of study participants CPR: cardiopulmonary resuscitation. * SPIKES, ABCDE, and BREAKS are global protocol names for breaking bad news.

Variable	Category	n (%)
Participants gender	Female	55 (40.4%)
	Male	81 (59.6%)
Participants job title	Ambulance paramedic	4 (2.9%)
	General practitioner (MD)	51 (37.5%)
	Nurse	27 (19.9%)
	Core training doctor	14 (10.3%)
	Pharmacist	7 (5.1%)
	Specialist	21 (15.4%)
	Other	12 (8.8%)
Participant working area	Emergency ward or department	40 (29.4%)
	Medical ward	27 (19.9%)
	Obstetrics and gynecology ward	4 (2.9%)
	Other	15 (11.0%)
	Pediatric ward	12 (8.8%)
	Primary health care centers	7 (5.1%)
	Surgical ward	18 (13.2%)
	Outside the hospital (Pharmacy, private clinic)	13 (9.6%)
How did the participant attempt to save the patient's life?	Giving orders to the team	9( 6.6%)
	Involved in the CPR	71 (52.2%)
	Supportive (Face mask/ Oxygen/ Cannulation/ Suction)	17 (12.5%)
	Observing vitals	8 (5.9%)
	Observing the team only	7 (5.1%)
	Nothing	16 (11.8%)
	Other	8 (5.9%)
How did the participant feel when the patient died?	Neutral	41 (30.1%)
	Sad	91 (66.9%)
	Other	4 (2.9%)
How did the participant report the death news to the family?	Directly (without any scenario or protocol)	45 (33.1%)
	Using any scenario or protocol	91 (66.9%)
Where did the participant learn to deal with reporting death?	From previous experiences	73 (55.3%)
	Medical school	31 (23.5%)
	Colleagues	32 (24.2%)
	The Internet or external sources	21 (15.9%)
Was the participant trained on any breaking bad news protocols?	SPIKES	29 (21.3%)
	Other (BREAKS, ABCDE, etc.)	0 (0.0%)
	None	107 (78.7%)
Was it the first time for the participant to report a death?	No	92 (67.6%)
Do participants believe that using protocols for breaking the news of death to the family may help them cope?	Yes	120 (88.2%)
Is delivering the news of death still as heavy and difficult as the first time?	Yes	89 (65.4%)
Do the participants feel that they have improved in reporting death?	Yes	104 (76.5%)
Do participants believe that training is necessary for dealing with reporting death?	Yes	88 (46.7%)

Table 3 presents the sociodemographic and other characteristics of the study’s deceased patients and their families. The mean approximate deceased patients' age was 47.27 ±26.47, and most of them were males (61.8%). In the participant's opinion, most deceased patients passed away in the emergency ward (36.0%), and cardiorespiratory arrest was the leading cause of death (35.3%) in the participants' views. The first person informed of death was the father or mother (27.2%), followed by the daughter or son (26.5%). Most often, the death was sudden and unexpected for the family (37.5%), and the informed individual or people cried upon hearing the news (61.8%).

**Table 3:** Sociodemographic and other characteristics of the deceased patients and their families. Abbreviations: SIDS: Sudden infant death syndrome, ICU: Intensive care unit

Variable	Category	n (%)
Deceased person gender	Female	52 (38.2%)
	Male	84 (61.8%)
Where did the deceased person lose his life?	Inpatient department	31 (22.8%)
	ICU	28 (20.6%)
	Emergency department	49 (36.0%)
	Outside the hospital	16 (11.8%)
	Other	12 (8.8%)
What caused the death, in your opinion?	Cardiorespiratory arrest	48 (35.3%)
	Brain complications (stroke, hemorrhage, etc.)	6 (4.4%)
	Abdominal complications (obstruction, perforation, aneurysm, etc.)	3 (2.2%)
	Arrived dead	18 (13.2%)
	Post-operative complications	11 (8.1%)
	Respiratory complication (Pulmonary embolism, hemorrhage, etc.)	14 (10.3%)
	Shock (septic, anaphylactic, neurogenic, etc.)	15 (11.0%)
	Suicide	2 (1.5%)
	Syndromic/Birth complication/SIDS	7 (5.3%)
	Trauma (road traffic accident, gunshot, falling, etc.)	7 (5.1%)
	Other	5 (3.7%)
What was the extent of the family’s expectation of the death?	Expected	44 (32.4%)
	Sudden or unexpected	51 (37.5%)
	Some expected, others not	38 (27.9%)
	Other	3 (2.2%)
How did the family react when they were informed about the death?	Anxious	14 (10.3%)
	Broke the surroundings	5 (3.7%)
	Cried	84 (61.8%)
	Fainted	6 (4.4%)
	Normal	12 (8.8%)
	Shout	12 (8.8%)
	Other	3 (2.2%)
Who did you inform about the death first?	Brother/ Sister	25 (18.4%)
	Daughter/Son	36 (26.5%)
	Father/Mother	37 (27.2%)
	Wife or Husband	9 (6.6%)
	The whole family	6 (4.4%)
	Other	23 (16.9%)
Did the family accept the death in the view of the participant?	Yes	86 (63.2%)

Correlation of SPIKES score with participant age and clinical experience

Table 4 presents the Pearson correlation between study participants' age and occupational characteristics and the SPIKES Score used in the reported deaths. Age (p-value = 0.015), years of clinical experience (p-value = 0.021), and the number of times participants reported death (p-value = 0.001) are positively correlated with the SPIKES score used in the reported deaths. In other words, an increase in any of the mentioned participants' characteristics increased the SPIKES score used in death reporting.

**Table 4:** Pearson correlation between the SPIKES Score and the characteristics of study participants (medical staff) Correlation is significant at the 0.01 level (2-tailed). ** Correlation is significant at the 0.05 level (2-tailed). * Abbreviations: n=Number, Sig.= Significance. * SPIKES is a global protocol for breaking bad news.

		Age	Years of experience	Previous experience of reporting death news.	SPIKES Score
Participant’s age	Pearson Correlation	1	0.918^**^	0.585^**^	0.237^*^
	Sig. (2-tailed)		<0.001	<0.001	0.015
	N	136	136	92	136
Participants’ years of clinical experience	Pearson Correlation	0.918^**^	1	0.510^**^	0.226^*^
	Sig. (2-tailed)	<0.001		<0.001	0.021
	N	136	136	92	136
Participants’ experience of reporting death news.	Pearson Correlation	0.585^**^	0.510^**^	1	0.376^**^
	Sig. (2-tailed)	<0.001	<0.001		0.001
	N	92	92	92	92
SPIKES Score	Pearson Correlation	0.237^*^	0.226^*^	0.376^**^	1
	Sig. (2-tailed)	0.015	0.021	0.001	
	N	136	136	92	136

Logistic regression models between the family acceptance of death and the other variables

Table 5 presents the association between family acceptance of death and the participants' sociodemographic and occupational characteristics. The analysis found that participants who reported a higher number of deaths were more likely to achieve higher family acceptance of death (P-value= 0.031) than those who reported less. At the same time, there was no statistically significant correlation between the SPIKES score used in that death and family acceptance of it. Participants who didn’t feel that reporting death was still as heavy and difficult as the first time (AOR= 3.92, P-value= 0.014) were about four times more likely to gain family acceptance than those who felt so. Participants who worked in the medical ward were about seven times (AOR= 6.86, P-value= 0.020) more likely to gain family acceptance. We also found that family members are more likely to accept death if it is reported by a medical staff who was involved in the CPR process (AOR= 19.34, P-value = 0.046).

**Table 5:** Binary logistic regression model of the relation between family acceptance of death with the sociodemographic and occupational characteristics of the participants The reference category is: *No*. Abbreviations: AOR; adjusted odds ratio, CI; Confidence interval, Ref: Reference.

Variable	Category	P-value	AOR (95%CI)
Participant age (years)		0.662	0.94 (0.714,1.239)
Participant gender	Male	Ref.	
	Female	0.351	0.53 (0.141, 2.002)
Participant clinical experience (in years)		0.718	0.95 (0.698, 1.281)
The number of times participants reported the death.		0.031	1.04 (1.003, 1.071)
Participant job title	Core training doctor	Ref.	
	General practitioner (MD)	0.116	2.73 (0.780, 9.531)
	Nurse	0.717	1.28 (0.342, 4.751)
	Other	0.951	1.05 (0.220, 5.003)
	Pharmacist	1	1.00 (0.160, 6.255)
	Specialist	0.409	0.56 (0.143, 2.206)
	Ambulance paramedic	0.277	0.25 (0.021, 3.041)
Participant location while preserving the deceased's life.	Outside the hospital (Pharmacy, private clinic)	Ref	
	Medical ward	0.02	6.86 (1.355, 34.705)
	Obstetrics and gynecology ward	0.328	0.29 (0.023, 3.523)
	Other	0.743	1.29 (0.286, 5.774)
	Pediatric ward	0.848	0.86 (0.178, 4.126)
	Primary health care centers	0.888	1.14 (0.179, 7.283)
	Surgical ward	0.606	0.69 (0.164, 2.874)
	Emergency ward or department	0.375	1.78 (0.497, 6.374)
Participant involvement in preserving the deceased's life.	Observing the team only	Ref	
	Involved in the CPR	0.046	19.34 (1.056, 353.916)
	Nothing at all	0.378	4.19 (0.175, 99.523)
	Observing vital signs	0.382	0.17 (0.003, 8.977)
	Other	0.184	11.46 (0.313, 419.601)
	Supportive management	0.762	1.62 (0.072, 36.556)
	Giving orders to the team	0.368	4.23 (0.183, 97.638)
Is delivering the news of death still as heavy and difficult as the first time?	Yes	Ref	
	No	0.014	3.92 (1.322, 11.632)
Participant's SPIKES score in that death		0.102	0.64 (0.380, 1.091)

Table 6 presents the association between family acceptance of death with the sociodemographic and other characteristics of the deceased person and the family. The analysis found that the family is more likely to accept death if the patient was in the inpatient department (AOR= 16.01, P-value=0.028) than in the emergency department. Similarly, they were more likely to accept death if they had all expected it (AOR=15.32, P-value=0.001) or some of them expected it (AOR=5.42, P-value=0.023), as opposed to unexpected or sudden death. At the same time, the death of patients aged between 1-10 years (AOR= 0.08, P-value=0.006) or 20-30 years (AOR=0.11, P-value=0.002) was less likely to be accepted by the family compared to those aged above 70 years old.

**Table 6:** Binary logistic regression model of the relation between family acceptance of death with the sociodemographic and other characteristics of the deceased person and the family The reference category is: No. Abbreviations: AOR; adjusted odds ratio, CI; Confidence interval, Ref: Reference.

Variable	Category	P-value	AOR (95%CI)
The area where the patient lost his life	Emergency department	Ref	
	Intensive care unit (ICU)	0.903	1.13 (0.164, 7.759)
	Other	0.898	1.17 (0.106, 12.874)
	Outside the hospital	0.169	5.73 (0.476, 69.053)
	Hospital ward	0.028	16.00 (1.348, 190.101)
Deceased person age group	Age >70	Ref	
	Age group <1	0.093	0.29 (0.068, 1.229)
	Age group (1-10)	0.006	0.08 (0.013, 0.487)
	Age group (10-20)	0.194	0.32 (0.058, 1.778)
	Age group (20-30)	0.002	0.11 (0.025, 0.452)
	Age group (30-40)	0.254	0.42 (0.096, 1.856)
	Age group (40-50)	0.084	0.24 (0.048, 1.211)
	Age group (50-60)	0.544	1.69 (0.310, 9.212)
	Age group (60-70)	0.103	0.39 (0.123, 1.211)
Gender of the deceased person	Male	Ref	
	Female	0.458	1.77 (0.393, 7.958)
What were the expectations of the family regarding the death of the patient?	Sudden or unexpected	Ref	
	Some expected, others not	0.023	5.41 (1.258, 23.313)
	Other	0.982	0.96 (0.024, 37.752)
	Expected	0.001	15.32 (2.888, 81.281)
Who was the first person informed of the death?	The whole family	Ref	
	Brother	0.263	0.27 (0.026, 2.699)
	Daughter	0.887	0.80 (0.037, 17.196)
	Mother	0.152	0.17 (0.014, 1.938)
	Other	0.223	0.23 (0.021, 2.456)
	Sister	0.278	0.20 (0.011, 3.661)
	Son	0.748	0.69 (0.068, 6.882)
	Uncle or Aunt	0.708	0.60 (0.041, 8.732)
	Father	0.265	0.27 (0.028, 2.676)
	Wife or Husband	0.28	0.25 (0.020, 3.100)

## Discussion

Discussion

Several studies have reported a substandard quality of care and miscommunication in Palestine [21-24]. The death notification process is generally regarded as onerous, demanding, and challenging by the medical staff member [17,25]. Breaking death news is one of the major elements of medical communication and it should be carried out under the most appropriate conditions to reduce both the pain of the relatives and the stress they experience [26,27]. In this study, we assessed the death reporting skills of Palestinian healthcare providers along with analyzing factors that impact the family's acceptance of death. Also, we assessed the knowledge and eligibility of using the global protocol (SPIKES) in the Palestinian population.

Our sample indicates that almost all types of medical careers are vulnerable to reporting death at some point in their life. The analysis showed that even though the majority (88.2%) of participants believed that using protocols for breaking the news of death to the family may help them cope, only a minority (21.3%) of them have received training on these protocols. Better education can also help people overcome their fears, including but not limited to fear of being blamed, not knowing what to say, being unable to handle the reactions of family members, or being unable to articulate their sentiments or concerns [13,28]. Therefore, training programs and workshops should be done to improve the knowledge and use of breaking bad news protocols for all medical personnel.

As shown in Table 4, some associations between family acceptance of death and the participants' sociodemographic and occupational characteristics were found. The analysis showed that participants who have reported a higher number of deaths are more likely to achieve family acceptance of death than those who have reported fewer deaths. These findings contradict with those who argue that participants who have dealt with a few deaths are superior at telling family members about death [7,8,29]. Participants who have not felt that reporting death is still as heavy and difficult as the first time are about four times more likely to gain family acceptance than those who lacked the confidence and experience. Lack of experience or confidence, apprehension, and distress negatively implicated the death reporting process, as shown by several studies [15-17,30,31]. Limited time and lack of resources also could be a barrier to using death reporting protocols and skills [32]. We recommend that inexperienced workers observe as much as possible death reporting by experienced workers, followed by gradually involving them, to improve their death reporting skills and increase their confidence. 

Participants who worked in the medical ward were about seven times more likely to gain family acceptance. This is attributed to the fact that communication between the medical staff and the patient’s families is facilitated when rapport and bonds between them are acknowledged and established during the patient’s hospital stay. On the other hand, the staff working in the medical inpatient department are usually more experienced in palliative and hospice care, and a significant part of the medical ward admissions are terminal cases. These results are in line with other studies [33,34]. Thus, we recommend involving such experienced staff in unexpected or young-aged death reporting.

Our analysis revealed that age, years of clinical experience, and the number of participant reports of death are positively correlated with having a higher SPIKES score. This could be attributed to medical practitioners’ communication skills than the framework itself. In other words, the older and more experienced the care provider, the better they will be at breaking death news, especially if they have reported many deaths news before. However, this study has no statistically significant correlation between the SPIKES score and family acceptance. Even though most of the current literature advises using breaking bad news protocols, some studies have postulated that despite the protocols’ widespread use, their shortcomings are openly acknowledged, and they also question whether patients benefit from them [25,30,32,35,36]. On the other hand, our study indicates that family members are more likely to accept death if it is reported by a medical staff who was involved in the CPR process. Additionally, several studies have found that letting family members watch CPR lessened death-related pain [37-39]. Further research is recommended regarding the benefit of using such protocols on the Palestinian and Arab populations. This could be attributed to the fact that members who are involved in CPR are usually experienced and more vulnerable to death reporting. Also, family acceptance may increase as a result of their belief that the staff members did their best trying to save the deceased person's life. We suggest allowing families to observe CPR without interfering with the medical staff. It is preferable that a member of that staff should report the death, as it leads to higher family acceptance. 

The language used to convey the bad news, the person breaking it, the location where it is delivered, and the method are just a few of the variables that can affect those who cope with one of the most difficult and stressful times in their lives [30,40]. Our analysis shows that the family is more likely to accept death if the patient has been admitted to the hospital ward compared to the emergency department. This is most likely because most deceased people spend some time in an inpatient department than in an emergency department, and breaking death news is seldom taught to emergency medical services personnel. Also, emergency department workers may not have enough time to prepare for the proper notification process [32]. Similarly, families were more likely to accept the death if they have expected it or some of them expected it as opposed to unexpected or sudden death. It is argued that the way the family members are treated will change depending on whether the patient is already dead or still alive and being given resuscitation [15]. Several studies have reported that it is easier to report death if the decedent passes away following a protracted, well-documented illness or after an autopsy than if s/he dies suddenly or unexpectedly [41-43]. Besides, the death of patients aged between 1-10 years or 20-30 years is less likely to be accepted by the family compared to those aged above 70 years old. It is known that child death is more devastating than other familial deaths, even though it is not uncommon in Muslim countries like Palestine for people to accept death at an early age due to religious beliefs [44-46]. More plans and strategies are needed to ease delivering bad news for unexpected or young death cases, including arranging the adequate time and appropriate place for that. Additionally, communication training programs should focus on emergency department workers who are more vulnerable to dealing with such situations.

Limitations of the study

The study's main notable limitation is that all participants are from Palestine and can't be generalized to the whole Arab population. Another challenge is the disproportionate sample size of different medical fields, as some medical careers have a higher probability of death reporting than others. Some may have poor recall of the details of the last death news breaking experience. Family acceptance of death was determined by the participants, which makes it subjective and highly dependent on participant perspectives. Even though we provided clear instructions for each question and mandated a compulsory revision at the end of the survey, the nature of an online survey may introduce bias by encouraging people to fill out the information quickly and improperly. Additionally, some medical professionals struggle to use current technologies (mostly older generation), which makes completing the questionnaire challenging. Others do not use social media, making reaching these groups difficult. Even though quantitative designs are frequently better at encapsulating numerical data to investigate a reality or phenomenon in a given population, a thorough qualitative supplement would be necessary to fully comprehend any results and generalize findings.

## Conclusions

Given that our sample includes almost all medical specialties who reported death at some point in their lives, our findings suggest that almost all Palestinian medical health professionals need to be given training in establishing a rapport with patients and their families, and in dealing with death situations and breaking bad news. A medical professional's communication abilities and skills are more crucial than familiarity with the SPIKES framework in Palestine. Our research shows that when a medical team member performing CPR reports a death, family members are more likely to accept it. Our study findings show that families are less likely to accept death in the unexpected or young age categories. This suggests that greater care and attention should be given when reporting such deaths. Additionally, reporting a death in an emergency department should be done with greater care, and the family should be spared as much grief as possible. Subsequently, training should be focused on staff who work in the emergency department and those who deal with young age groups or sudden deaths. Finally, further research is recommended to develop a better death reporting approach for the Palestinian population or even the Arab one.
